# Assessing *Anopheles* vector species diversity and transmission of malaria in four health districts along the borders of Côte d’Ivoire

**DOI:** 10.1186/s12936-021-03938-1

**Published:** 2021-10-18

**Authors:** Firmain N. Yokoly, Julien B. Z. Zahouli, Graham Small, Allassane F. Ouattara, Millicent Opoku, Dziedzom K. de Souza, Benjamin G. Koudou

**Affiliations:** 1 Unité de Formation et de Recherche Sciences de la Nature, Université Nangui Abrogoua, Abidjan, Côte d’Ivoire; 2 Centre Suisse de Recherches Scientifiques en Côte d’Ivoire, Abidjan, Côte d’Ivoire; 3 Centre d’Entomologie Médicale et Vétérinaire, Université Alassane Ouattara, Bouaké, Côte d’Ivoire; 4 Innovative Vector Control Consortium (IVCC), Pembroke Place, Liverpool, L3 5QA UK; 5 Department of Parasitology, Noguchi Memorial Institute for Medical Research, University of Ghana, Legon, Accra, Ghana; 6 European & Developing Countries Clinical Trials Partnership, Cape Town, South Africa

**Keywords:** Malaria, *Plasmodium*, *Anopheles*, Border health district, Côte d’Ivoire

## Abstract

**Background:**

Although malaria and *Anopheles* mosquito vectors are highly prevalent in Côte d’Ivoire, limited data are available to help understand the malaria vector density and transmission dynamics in areas bordering the country. To address this gap, the *Anopheles* mosquito species diversity, the members of the *Anopheles gambiae* complex and the transmission of malaria were assessed in four health districts along the borders of Côte d’Ivoire.

**Methods:**

From July 2016 through December 2016 and July 2017 through December 2017, adult *Anopheles* mosquitoes were collected in four health districts of Côte d’Ivoire (Aboisso, Bloléquin, Odienné and Ouangolodougou) using standardized window exit trap (WET) and pyrethrum knockdown spray collection (PSC) methods. The collected mosquitoes were identified morphologically at species level and the members of the *An. gambiae* complex were separated using short interspersed nuclear element-based polymerase chain reaction **(**SINE-PCR). *Anopheles gambiae *sensu lato* (s.l.)*, *Anopheles funestus s.l.* and *Anopheles nili* specimens were analysed for malaria *Plasmodium* parasite detection using the cytochrome oxidase I gene (COX-I), and malaria prevalence among human population through local Ministry of Health (MoH) statistical yearbooks.

**Results:**

A total of 281 female *Anopheles* were collected in Aboisso, 754 in Bloléquin, 1319 in Odienné and 2443 in Ouangolodougou. Seven *Anopheles* species were recorded including *An. gambiae s.l.* (94.8–99.1%) as the main vector, followed by *An. funestus s.l.* (0.4–4.3%) and *An. nili* (0–0.7%). Among *An. gambiae s.l*., *Anopheles coluzzii* represented the predominant species in Aboisso (89.2%) and Bloléquin (92.2%), while *An. gambiae *sensu stricto (*s.s*.) was the major species in Odienné (96.0%) and Ouangolodougou (94.2%). The *Plasmodium* sporozoite infection rate in *An. gambiae s.l.* was highest in Odienné (11.0%; n = 100) followed by Bloléquin (7.8%, n = 115), Aboisso (3.1%; n = 65) and Ouangologoudou (2.5%; n = 120). In *An. funestus s.l*., *Plasmodium falciparum* sporozoite infection rate was estimated at 6.2% (n = 32) in Bloléquin, 8.7% (n = 23) in Odienné. No *An. funestus s.l.* specimens were found infected with *P. falciparum* sporozoite infection in Ouangolodougou and Aboisso. No *P. falciparum* sporozoite was detected in *An. nili* specimens in the four health districts. Among the local human populations, malaria incidence was higher in Odienné (39.7%; n = 45,376) and Bloléquin (37.6%; n = 150,205) compared to that in Ouangolodougou (18.3%; n = 131,629) and Aboisso (19.7%; n = 364,585).

**Conclusion:**

*Anopheles* vector species diversity, abundance and *Plasmodium* sporozoite infection were high within the health districts along the borders of the country of Côte d’Ivoire, resulting in high malaria transmission among the local populations. *Anopheles gambiae s.l*. and *An. funestus s.l.* were found to be highly infected with *Plasmodium* in the health districts of Bloléquin and Odienné where higher malaria incidence was observed than the other districts. This study provides important information that can be used to guide Côte d’Ivoire National Malaria Control Programme for vector control decision-making, mainly in districts that are at the country borders.

## Background

Malaria remains the deadliest tropical infectious disease, with higher incidences in Africa [1]. In 2018, malaria has caused over 228 million cases and 405,000 deaths worldwide, of which 93% has occurred in sub-Saharan Africa [1]. In 2019, the overall number of cases increased from 228 to 229 million of malaria cases with 409,000 deaths [2]. In Côte d’Ivoire, malaria is still a major public health challenge and the leading reason for consultations in health services. It is responsible for up to 43% of morbidity, 11.8% of mortality, 40% of school absenteeism, 50% of loss of agricultural income and 62% of hospitalizations [3]. According to the world malaria report, the entire Ivorian population is at risk of malaria, with the most vulnerable being children under 5 years of age and pregnant women [4].

Malaria transmission in Africa is very heterogeneous due to eco-climatic variations across the continent [5]. Currently, five species of the *Plasmodium* parasites have been identified as being responsible for malaria infection in humans [6, 7]. Of these, *Plasmodium falciparum* remains the most prevalent and most virulent species causing the deadly forms of malaria [7, 8]. The *Plasmodium* species responsible for human malaria are mainly transmitted by primary vector species, such as *Anopheles gambiae *sensu lato (*s*.*l*.) [9], *Anopheles funestus* group and *Anopheles nili* group [10–12].

In West Africa, two molecular forms have been identified in *An. gambiae s.l.*, formerly known as M and S. Recently, they have been identified as distinct species belonging to the *An*. *gambiae* complex and named *Anopheles coluzzii* for the M form and *An. gambiae *sensu stricto (*s.s*.) for the S form [10]. These species display strong anthropophilic host-seeking behaviour and longevity, causing large numbers of malaria cases [13]. *Anopheles* larvae are aquatic and found in a variety of breeding habitat types in terms of size, permanence, vegetation and water cleanliness [14]. Overall, the larvae of *An. gambiae s.l.* grow in small, shallow, relatively clean and sunny water reservoirs (puddles of water, stagnant water) [15, 16], and is more frequent and rain-dependent. *Anopheles coluzzii* is associated to permanent breeding sites and those resulting from human activity and prefers urban water collections and adapts quickly to pollution [17, 18]. According to Mourou et al. [19], the breeding habitats of this species are known to increase in number and productivity during the rainy season, but almost disappear during the dry season. Deploying major vector control interventions, such as the scale-up of long-lasting insecticidal nets (LLINs) and indoor residual spraying (IRS), requires detailed understanding of the species composition, distribution and behaviour dynamics of the local vectors to avoid limited impact or intervention failure [20, 21].

In Côte d’Ivoire, the malaria vectors *An. gambiae s.s.* and *An. coluzzii* are widespread across the country [22, 23], whilst *An. funestus s.l.* and *An. nili* act as secondary vectors [24]. *Anopheles gambiae s.s.* and *An. coluzzii* are well-adapted to diverse types of breeding sites (e.g., permanent breeding sites or temporary rain pools such as puddles, shallow wells, footprints, or in rice and vegetable fields), which are generally frequent in both rural and urban areas [25]. The country shows considerable bio-climatic variations from the North to the South, leading to the subdivision of the country into different ecological zones [26]. Thus, the South-East of Côte d’Ivoire is marked by coastal inland lagoons [27]. The southern region, especially the South-West, is covered with dense tropical rainforest. The Guinean forest-savannah mosaic belt extends across the middle of the country from the East to the West and the northern part belongs to the Sudanian savannah. All these ecological conditions contribute to the proliferation of many malaria vector species across the country [28]. This contributes to the stability of malaria transmission throughout the year, with peaks during the rainy season [29]. Identifying these vector species and their involvement in malaria transmission in these different ecological zones could provide essential information that could guide the planning and implementation of vector control measures. Furthermore, limited entomological data are available on disease transmission dynamics, particularly in districts along the borders of the country. This study assessed the species diversity of *Anopheles* mosquito vectors, members of the *An. gambiae* complex and transmission of malaria in four health districts at the border areas of Côte d'Ivoire.

## Methods

### Study sites

**Fig. 1:**
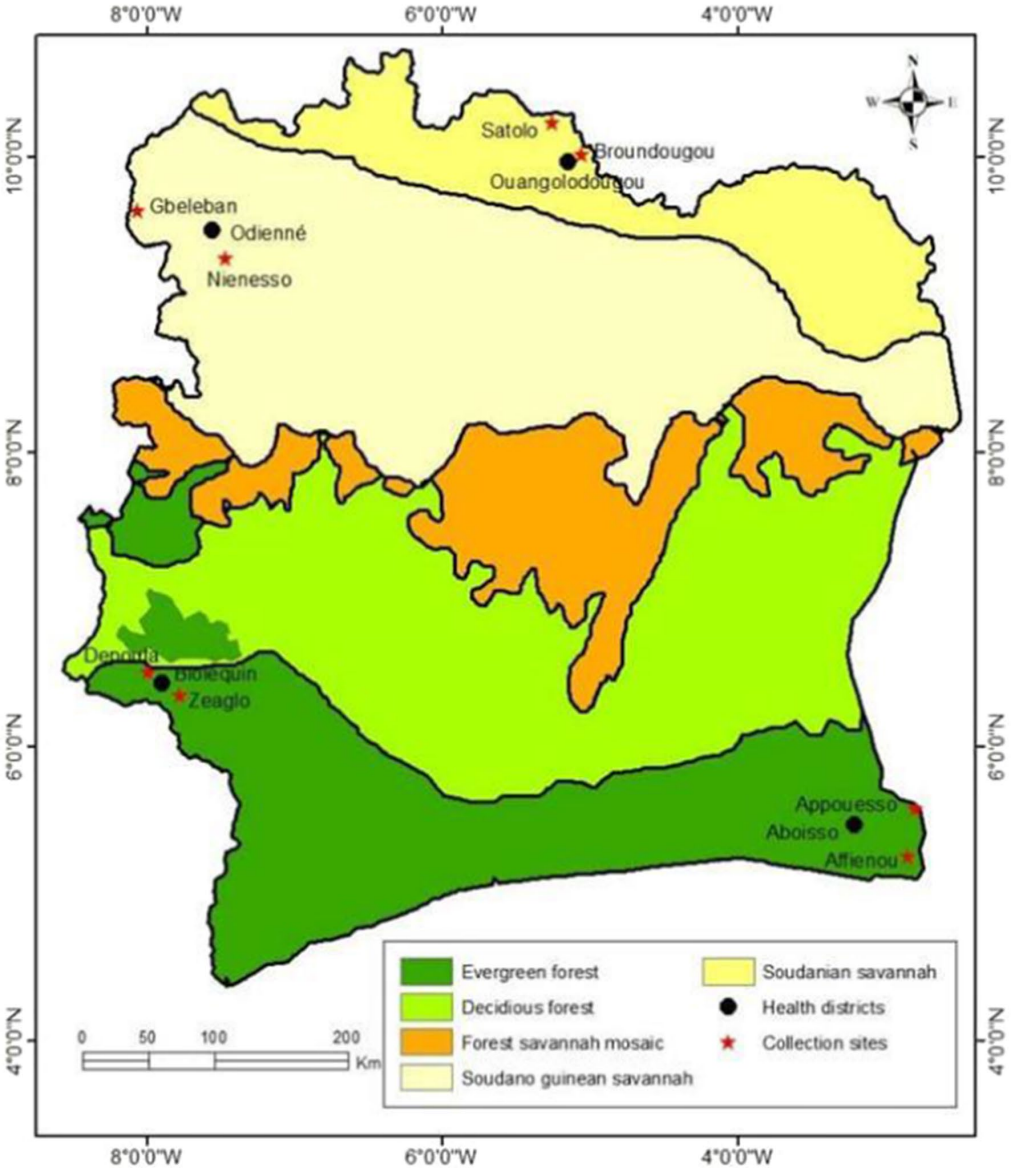
Map of Côte d’Ivoire showing the study sites in the four health districts (Aboisso, Bloléquin, Odienné and Ouangolodougou) along the borders of Côte d’Ivoire

The study was conducted in the four-health districts of Côte d’Ivoire. The health districts are namely Aboisso (5° 28′ N, 3° 12′ W) and Bloléquin (6° 34′ N, 8° 00′ W) Odienné (9° 30′ N, 7° 33′ W) and Ouangolodougou (9° 58′ N, 5° 09′ W) (Fig. 1).

The district of Aboisso is located in the primary rainforest of Côte d’Ivoire in the south-western border with Ghana. The climate is humid tropical, characterized by abundant rainfall with a mean annual of about 1500 mm of rain. The average annual temperatures are between 25 and 27 °C. This district covers an area of over 4662 km^2^ with a population of 307,852 people representing a density of about 66 inhabitants per km^2^ [30]. In the district of Aboisso, *Anopheles* mosquitoes were collected in the villages of Affiénou (5° 25′ N; 2° 56′ W) and Appouesso (5° 57′ N; 3° 10′ W). Both villages are irrigated by numerous streams and lowlands, and have modern and traditional housing, a health centre, electricity and water supply. Coffee, cocoa, rubber and palm oil are the main cash crops while vegetable, cocoyam and banana are the main food crops in the area.

The district of Bloléquin is situated in the dense forest zone in the West of Côte d’Ivoire at the border with Liberia. The population is about 123,336 inhabitants [30]. The climate is mountainous, with annual average rainfall sometimes exceeding 2000 mm per year and annual temperatures ranging from 15 to 33 °C. It covers an area of 2962 km^2^ with a population density of approximately 41 inhabitants per km^2^. The district is irrigated by important tributaries coming from the Sassandra and Cavally rivers, enabling the establishment of various permanent water bodies, puddles and small dams. The study sites in this district covered the villages of Zéaglo (6° 34′ N, 7° 47′ W) and Dépouta (6° 54′ N, 7° 57′ W). Only Zéaglo has a health centre, electricity, water supply and modern housing. The agricultural activity is mainly based on coffee, cocoa and rubber cultivation. Food crops are dominated by banana, cassava, maize and vegetables, and included flooded lowland rice paddy and rainfed rice cultivation.

The district of Odienné is in the savannah zone in the North-West of Côte d’Ivoire and shares a border in its western part with the Republic of Guinea. It covers an area of 14 000 km^2^ with a population of 193,364, giving a density 13.8 inhabitants per km^2^ [30]. The climate is tropical sub-humid with annual rainfall varying between 1400 and 1600 mm per year and annual temperatures ranging between 25.4 and 33 °C. The vegetation is essentially dominated by savannah type vegetation, with trees or shrubs with a grassy tendency. In some places, there are patches of forest and also forest galleries along the water bodies. The district is fed by tributaries of the Sassandra river such as the Bagoué and Tienba rivers. Our surveys were conducted in Gbéléban (09° 36′ N, 08° 08′ W) and Niénésso (09° 21′ N, 07°36′ W). Gbéléban is bordered at the South by the Gbanala river and has modern housing, a health centre, electricity and water, while Niénésso has no health centre and is bordered at the East by an undeveloped lowland which provides watering for cattle. Most of the local inhabitants are farmers and their staple crops include cereals, tubers, cotton and cashew nuts.

The health district of Ouangolodougou is in the savannah zone in the North of Côte d’Ivoire. It is bordered in the northern part by Burkina Faso and covers an area of 5380 km^2^, with an estimated population of 260,519 habitants, giving a density of 48.4 inhabitants per km^2^ [30]. The district is characterized by a Sudanese climate with a unimodal rainfall regimen from May to November. The annual rainfall varies from 1000 to 1400 mm, while the mean annual temperature ranges from 21 to 35 °C. The minimum temperatures can drop to 16 °C due to the Harmattan wind during December and January. The natural vegetation is mainly a mixture of savannah and open forest characterized by trees and shrubs that are approximately 8–15 m in height. The soil is highly conducive to agriculture and most of the local inhabitants are farmers with staple crops including rice, maize, and cotton. Rice is mainly cultivated during the rainy season in flooded soils. The study area included the villages of Broundougou (9° 59′ N; 05°09′95″ W) and Satolo (10° 10′ N; 05°27′ W). Broundougou has a health centre, electricity, water supply and modern houses, whilst Satolo has no modern infrastructure.

Before implementing the study, the local malaria prevalence from 2013 to 2015 recorded in the yearbooks in each district was explored to select the study sites.

### Study design

Entomological surveys were conducted in two villages in each of the four selected health districts of Côte d’Ivoire. The first phase of entomological collections was conducted the between July and December 2016 and a second phase of collections during July–December 2017 to capture seasonal variations in mosquito species diversity and abundance and malaria transmission over years.

### Adult mosquito collections

Adult mosquitoes were sampled using window exit traps (WETs) and pyrethrum knock-down spray collections (PSCs). In each site, 15 WETs were installed on the windows of inhabited houses for two consecutive days per survey. Mosquitoes trapped were collected every morning between 6 a.m. and 9 a.m. PSCs were performed early each morning from 6 a.m. and 8 a.m., before opening the windows, in ten rooms selected in different dwellings during 2 days per site per district. PSCs were performed in households that were different from those used for WET collections. In case of unavailability or refusal of participants, mosquitoes were collected from neighbouring houses.

### Field mosquito processing

*Anopheles* mosquitoes were sorted from collected culicines and morphologically identified using morphological identification keys [31, 32]. The ovaries of the females of *Anopheles* vectors (*An. gambiae s.l*., *An. funestus s.l*., and *An. nili*) were dissected for parity using the degree of coiling of ovarian tracheoles [33]. All collected *Anopheles* females were stored individually in Eppendorf tubes containing desiccant, labelled according to the study site, point and date of collection, and stored at − 20 °C for further molecular analysis in the laboratory at the Centre Suisse de Recherches Scientifiques en Côte d’Ivoire in Abidjan, Côte d’Ivoire.

### Molecular identification of *Anopheles gambiae* complex members

DNA was extracted from the legs of *An. gambiae s.l.* mosquitoes using the boiling method [34]. Briefly, three legs of each female *Anopheles* were cut and crushed in 100 mL of distilled water and boiled at 95 °C for 10 min. The supernatant was pipetted and transferred into new labelled Eppendorf tubes which were stored at − 20 °C and used as template for the polymerase chain reaction **(**PCR**)**.

*Anopheles gambiae* complex members were identified according to the SINE-PCR molecular method described by Santolamazza et al. [35]. The primer F6.1A with the sequence 5′-TCGCCTTAGACCTTGCGTTA-3′ was used to distinguish *An. coluzzii* and the primer R6.1B with the sequence 5′-CGCTTCAAGAATTCGAGATAC-3′ to distinguish *An. gambiae s.s*. A LongGene® thermocycler (A200 Gradient Thermal cycler; LongGene Scientific Instruments Co., Ltd Hangzhou, P.R. China) was used with the following programme: 37 °C for 30 min, 94 °C for 30 s, and 59 °C for 30 s; 72 °C for 1 min repeated 35 times; and a final step at 72 °C for 10 min to finish the reaction. An agarose gel was prepared with 2% agarose in TBE (Tris/borate/EDTA) containing ethidium bromide at 10 mg/ml. The PCR product was loaded onto the agarose gel and allowed to migrate under a voltage of 100 V for 70 min. The result was visualized with a UV illuminator (TOYOBO Trans Modele TM-20).

### Determination of sporozoite rates in *Anopheles gambiae*

DNA was extracted from the head and thorax of the adult females of *Anopheles* vector mosquitoes in each district [36] and screened for *Plasmodium* DNA using the fast COX-I PCR method described in Echeverry et al. [37]. This is a very sensitive and rapid method which uses a set of primers, COX-IF (5′AGAACGAACGCTTTTAACGCCTG 3′) and COX-IR (3′ ACTTAATGGTGGATATAAAGTCCATCCwGT 5′), to amplify a polymorphic fragment of the COX-(I) gene using a recombinant DNA polymerase. The thermocycler (TaKaRa PCR Thermal Cycler Dice TP600) program was 94 °C for 5 min followed by 40 cycles of 94 °C for 1 min, 62 °C for 1 min and 72 °C for 90 s, and a final elongation step at 72 °C for 10 min. Five microlitres of the PCR product was visualized on 1% agarose gel in order to confirm amplification of the expected ~ 540 bp product (*Plasmodium* genus positive).

### Malaria infection prevalence

In each health district, data on the malaria prevalence in the local populations were collected from the MoH statistical yearbooks. The total number of consultations was compared with the number of people examined with malaria from 2016 to 2017.

### Data analysis

Data were double entered in Microsoft Excel 2013 software and transferred to STATA 14 (Stata Corp, College Station, Tx, USA) for analysis. The Kruskal–Wallis (KW) test was used to compare the differences in mosquito densities. The parity rate (PR) was calculated as the number of parous females multiplied by 100 and divided by the total number of females dissected. The *P. falciparum* sporozoite infection rate in each vector species population was calculated by dividing the number of *Plasmodium*-positive mosquitoes by the total number of mosquitoes tested, and this was expressed as a percentage (%). The χ^2^ test was used to compare sporozoite and PR between the collection sites and the health districts. All differences were considered significant at *p* < 0.05.

## Results

### Mosquito species composition

**Fig. 2:**
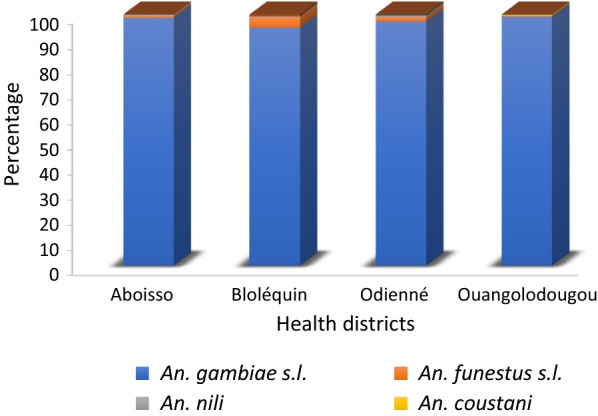
Species composition of *Anopheles* mosquitoes collected in four health districts of Côte d’Ivoire from July to December in 2016 and 2017

Figure 2 shows the species composition of vector species among the anopheline fauna collected in the four health districts of Aboisso, Bloléquin, Odienné and Ouangolodougou. During both year collection periods, a total of 4797 adult *Anopheles* females were collected using WET and PSCs. Of these, *An. gambiae s.l*., *An. funestus s.l*. and *An. nili* accounted for 99.4% of *Anopheles* collected. In Aboisso, Bloléquin, Odienné and Ouangolodougou districts, these three *Anopheles* species together accounted for 100% (n = 281), 99.2% (n = 748), 99.3% (n = 1309) and 99.5% (n = 2431) of anopheline fauna, respectively. *Anopheles gambiae s.l.* was predominant in all study sites, with particularly higher density in the northern districts of Odienné and Ouangolodougou in savannah area compared to the districts located in the forest area (χ^2^ = 22.85; df = 4, p < 0.001). *Anopheles funestus s.l*. was found in relatively low proportion in three of the four districts: Bloléquin 4.3% (n = 754); Odienné 1.8% (n = 1319); Aboisso 0.7% (n = 281); and Ouangolodougou 0.4% (n = 2443). *Anopheles nili* was not found in Ouangolodougou, though the number collected in the others three sites was very low.

### Distribution and parity rate of *Anopheles gambiae s.l.* in the four districts

**Fig. 3:**
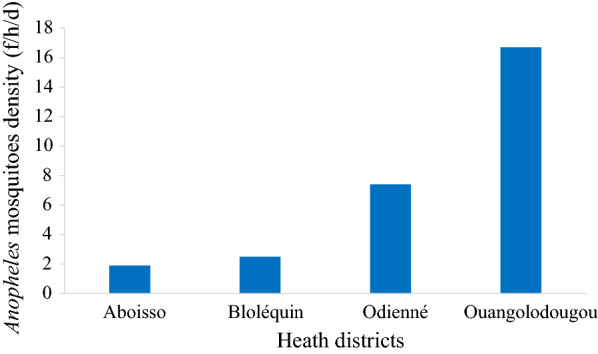
*Anopheles gambiae s.l.* density distribution in four health districts of Côte d’Ivoire from July to December in 2016 and 2017

*Anopheles* mosquito density varied significantly from one health district to another (KW = 11.03; df = 7, *p* = 0.012). The highest density of *An. gambiae s.l.* was observed in Ouangolodougou [16.7 females/household/day (f/h/d)] and the lowest in Aboisso (1.9 f/h/d). The average densities observed in Aboisso and Bloléquin were similar, being 1.9 f/h/d and 2.5 f/h/d, respectively. In Odienné, the average density was 7.4 f/h/d. *Anopheles gambiae s.l.* mosquito densities in the four districts were significantly different (KW test = 7.06; df = 3; *p* = 0.002) (Fig. 3).

Table 1 describes the parity rates of *An. gambiae s.l.* collected in the four health districts. The majority of *An. gambiae s.l.* dissected were old with an average parity rate of 56.7 (n = 4651). The highest parity rate was observed in *An. gambiae s.l.* in Odienné (69.0%; n = 1258), followed by Aboisso (63.8%; n = 276), Bloléquin (51.1%; n = 735), and Ouangolodougou (49.2%; n = 2382). The proportions of parous and nulliparous females of *An. gambiae s.l.* dissected over the collection period were significantly different in Odienné (χ^2^ = 6.46; df = 1; p = 0.01), and Ouangolodougou (χ^2^ = 15.30; df = 1; *p* < 0.001). Significant variation was observed in *An. gambiae s.l*. parity rates between health districts (*p* < 0.001).

**Table 1:** Parity rates of *Anopheles gambiae s.l.* collected in four cross-border districts of Côte d’Ivoire from July 2016 to December 2017

Districts	Total dissected	No. parous	Parity rate		*p-value* (Parous/nulliparous)
	n	n	(%)	95%CI (%)	
Aboisso	276	176	63.8	[57.7–69.4]	0.44
Bloléquin	735	420	57.1	[53.4–60.7]	0.78
Odienné	1258	868	69.0	[66.3–71.5]	0.01*
Ouangolodougou	2382	1172	49.2	[47.1–51.2]	< 0.001*
Total	4651	2636	56.7	[56.1–63.2]	0.31

*n* number, *CI* confidence interval

*Significant difference, *p* = p-value

### *Gambiae* complex species distribution

**Fig. 4:**
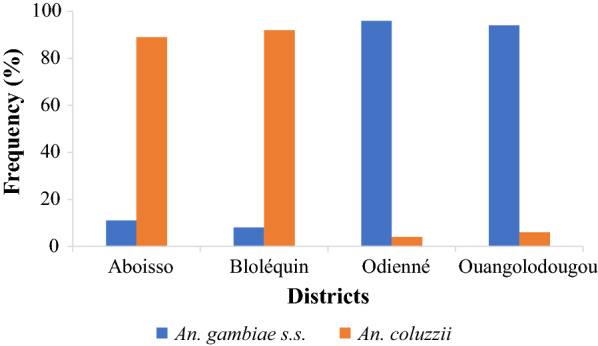
Distribution of members of *Anopheles gambiae* complex in four cross-border districts of Côte d’Ivoire from July to December in 2016 and 2017

Figure 4 illustrates the different *An. gambiae s.l.* species recorded in the study areas within the health districts of Aboisso, Bloléquin, Odienné and Ouangolodougou. All *An. gambiae s.l.* specimens analysed were either *An. gambiae s.s*. or *An. coluzzii*. However, the proportions of each species substantially varied among the health districts. *Anopheles coluzzii* was predominant in the health districts of Aboisso and Bloléquin located in the forest zone exhibiting respective proportion of 89.2% (n = 65) and 92.2% (n = 115) while *An. gambiae s.s.* exhibited a high frequency in the districts of Odienné (96.0%; n = 100) and Ouangolodougou (94.2%; n = 120) located in the savannah zone.

### Plasmodium infection rate in *Anopheles* mosquito species

Table 2 exhibits the *P. falciparum* sporozoite rates detected in the *Anopheles* species in the four health districts of Aboisso, Bloléquin, Odienné and Ouangolodougou. Only *An. gambiae s.l.* and *An. funestus s.l.* was found to be infected with *P. falciparum* sporozoites, while no *P. falciparum* sporozoites was detected in the collected *An. nili* specimens. In total, 29 mosquitoes were found infected, and the infection rate of malaria vectors ranged from 2 to 10%. Overall *P. falciparum* sporozoite rate in *An. gambiae s.l*. was 3.1% (n = 65, % CI 0.4–10.7) in Aboisso, 7.8% (n = 115, % CI 3.6–14.3) in Bloléquin, 11.0% (n = 100, % CI 5.6–18.8) in Odienné and 3.3% (n = 120, % CI 0.9–8.3) in Ouangolodougou. There was no significant difference in *P. falciparum* sporozoite rate in *An. gambiae s.l.* between the health districts (χ^2^ = 7.33; df = 3, p = 0.065). In *An. funestus s.l*., the *P. falciparum* sporozoite rate was 6.2% (2/32, % CI 0.7–20.8) in Bloléquin and 8.7% (2/23, % CI 1.1–28.0) in Odienné, but no *P. falciparum* sporozoites was detected in this species of Aboisso (0/2) and Ouangolodougou (0/11).

**Table 2:** *Plasmodium falciparum* sporozoite rates in *Anopheles* mosquito species collected in four cross-border districts of Côte d’Ivoire from July 2016 to December 2017

Species	Aboisso		Bloléquin		Odienné		Ouangolodougou	
	No. tested (infected)	SR (%) CI (95%)	No. tested (infected)	SR (%) CI (95%)	No. tested (infected)	SR (%) CI (95%)	No. tested (infected)	SR (%) CI (95%)
*An. gambiae s.l*	65 (2)	3.1 [0.4–10.7]	115 (9)	7.8 [3.6–14.3]	100 (11)	11.0 [5.6–18.8]	120 (3)	2.5 [0.5–7.1]
*An. funestus s.l*	2 (0)	0.0 [0.0–84.2]	32 (2)	6.2 [0.7–20.8]	23 (2)	8.7 [1.1–28.0]	11 (0)	0.0 [0.0–28.5]
*An. nili*	2 (0)	0.0 [0.0–84.2]	1 (0)	0.0 [0.0–97.5]	4 (0)	0.0 [0.0–60.2]	0.0 (0)	0.0 [0–0]
Total	69 (2)	2.9 [0.3–10.1]	148 (11)	7.4 [3.8–12.9]	127 (13)	10.2 [5.6–16.9]	131 (3)	2.3 [0.5–6.5]

*No. tested* number of mosquitoes tested, *SR* sporozoite rate, % percentage, *CI* confidence interval

### Malaria prevalence among local human populations

**Fig. 5:**
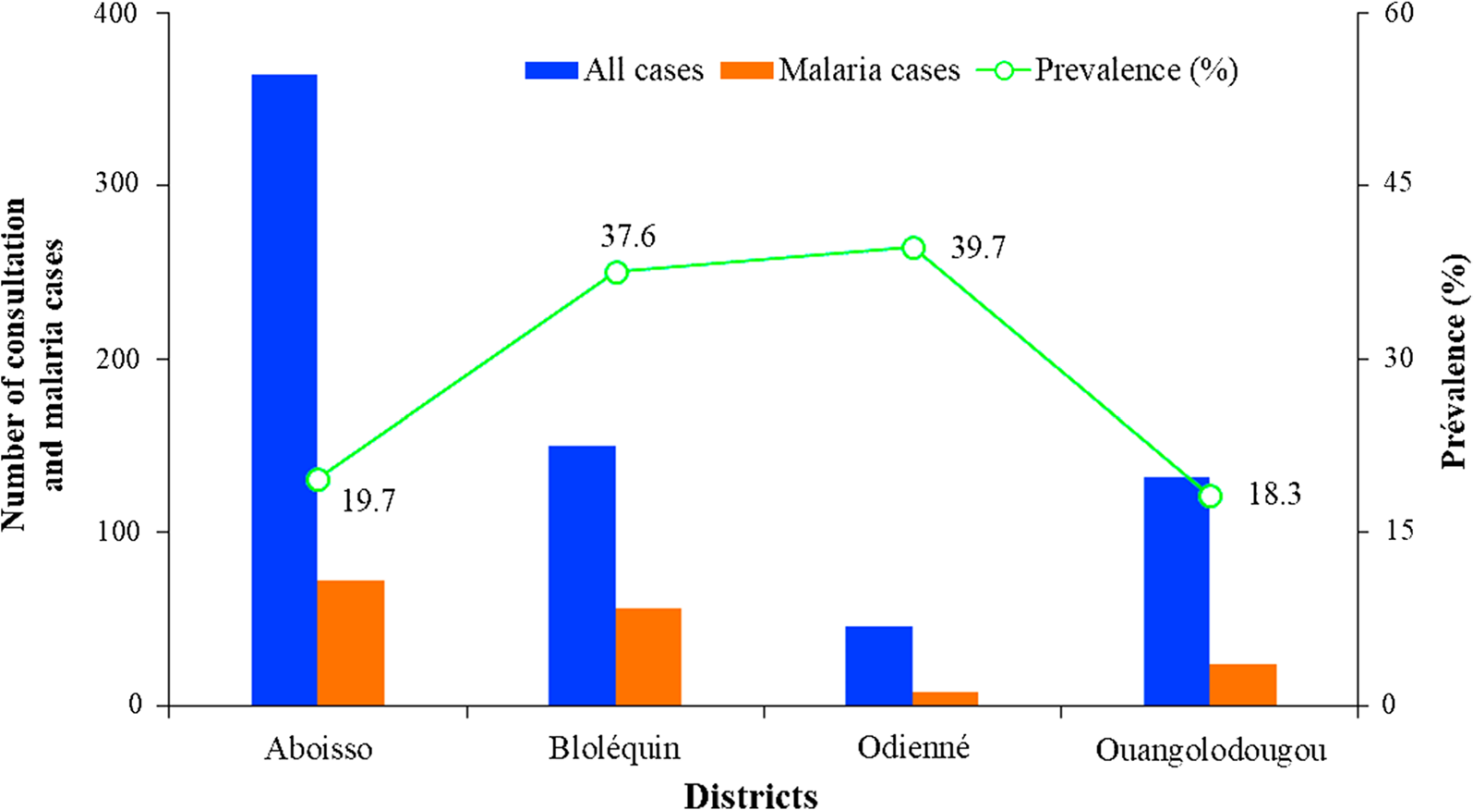
Prevalence of malaria among the population of the four health districts of Côte d’Ivoire from 2016 to 2017

Figure 5 indicates the malaria prevalence rate among the population of the four health districts from 2016 to 2017. The prevalence rate was high in Odienné (39.7%; n = 45,376) and Bloléquin (37.6%; n = 150,205) health districts, compared to Ouangolodougou (18.3%; n = 131,629), and Aboisso (19.7%; n = 364,585).

## Discussion

Malaria is still a leading cause of death and poverty in the majority of the countries in sub-Saharan Africa due to country vectors specificity and even differences within ecological zones of a country implying targeted monitoring to better understand and support vector control decision-making. This study assessed *Anopheles* mosquito vectors and malaria transmission in district health facilities at the borders of Côte d’Ivoire, coupled with the malaria incidence during the second semester of both 2016 and 2017 [38]. In this study, the relative diversity and abundance of *Anopheles* mosquitoes in four districts located at the borders of Côte d'Ivoire has been highlighted and assessed their implications in malaria transmission to local communities. The results have shown that *An. gambiae s.l.* was most the abundant vector of malaria in the four districts. Moreover, *Plasmodium* sporozoite infection rate in *An. gambiae s.l.* and *An. funestus s.l.* was higher in Odienné (11.0% and 8.27%, respectively) and Bloléquin (7.8% and 6.2%, respectively), compared with Aboisso (3.1% and 0%, respectively) and Ouangolodougou (2.5% and 0%, respectively). Similarly, malaria prevalence rate among people in Odienné (39.7%) and Bloléquin (37.6%) health districts was higher compared with that in Ouangolodougou (18.3%), and Aboisso (19.7%) health districts.

A diversity of *Anopheles* mosquito species including seven *Anopheles* species was recorded, among which three (*An. gambiae s.l.*, *An. funestus s.l.* and *An. nili*) have previously been incriminated in malaria transmission in Côte d’Ivoire [22, 39]. Moreover, *An. gambiae s.l.* was the predominant species in all study sites, with particularly higher abundance in the northern health districts of Ouangolodougou and Odienné located in savannah zones compared with the districts of Aboisso and Bloléquin situated in forest zones. The high diversity and variation in the relative abundance of *Anopheles* mosquito species might result from a combination of ecological and climatic factors favouring the larval development of some species. Indeed, permanent water sources, puddles and small dams are probably abundant in these areas [40, 41].

The diversity and variation in *Anopheles* mosquito species composition are mostly related to human activities (e.g., rice farming, vegetable crops) [42, 43]. Odienné and Ouangolodougou are rural districts in which several crops are cultivated including rice, maize, yam, vegetable crops, cashew nuts and cotton. Rice paddies and vegetable crops were strongly associated with high densities of malaria vectors [44, 45]. In addition, *An. gambiae s.l.* exhibited high parity rates in all districts, thus suggesting that a significant proportion of the local vector populations that have sufficient lifespan allowing for the completion of *Plasmodium* parasite lifecycle and transmission to humans. Similar findings have previously been reported in Côte d’Ivoire [46, 47].

This study showed that *An. coluzzii* was the predominant species in forest area, whilst *An. gambiae* s.s. was the dominant species in savannah. The relative abundance of these two species is associated with specific and characteristic breeding sites as previously reported in Côte d'Ivoire [40, 48]. The abundance of *An. coluzzii* in samples from forest areas could be related to the type of breeding sites and the climatic conditions in these study sites [49, 50]. Several studies carried out in Côte d'Ivoire have shown a predominance of *An. coluzzii* in forest area especially in the western [22] and south-eastern [51] parts. The highest abundance of *An. gambiae* s.s. was observed in the savannah area where relatively abundant precipitation provides more favorable humidity and temperature conditions [52, 53]. The predominance of *An*. *gambiae s.s.* in savannah zones has been observed by Touré et al*.* [54] and Tia et al*.* [48], suggesting that environmental conditions in savannah zones are unfavourable for the reproduction and the survival of *An. coluzzii*.

Understanding malaria transmission at the local level is essential for the development and implementation of effective vector control strategies. Thus, to identify potential vector species of malaria in our various study sites, individual females of *An. gambiae s.l.* were tested using molecular PCR for the presence of *P. falciparum* sporozoites. The finding showed that only two species were involved in malaria transmission across the four health districts, with *An. gambiae s.l.* being the main malaria vector in all districts. In addition, *An. gambiae s.l.* and *An. funestus s.l.* were efficient vectors of *P. falciparum* in these two cross-border districts. This observation is consistent with findings from previous studies showing that the high capacity of malaria vectors ensures high transmission in an ecological area is intimately related with environmental conditions [21, 22]. Although infection rates were almost similar between study sites, the intensity of transmission was very heterogeneous. Malaria transmission is lower in Aboisso and Ouangolodougou health districts. In contrast, it remains relatively high in Bloléquin and Odienné districts where two additional *Anopheles* vectors are involved in malaria transmission. This high transmission of malaria could possibly be due to the high infection rate of malaria vectors and the non-respect of vector control measures in these districts where control strategy is based mainly on the distribution of distribution of LLINs. In addition, the presence of several vectors in the same area could significantly increase the risk of malaria transmission. This study suggests that malaria vector control interventions should be strengthened in Bloléquin and Odienné in order to reduce or eliminate the burden of malaria in these districts.

The low prevalence of malaria observed (< 20%) in the health districts of Aboisso and Ouangolodougou suggests that both districts are in areas of moderate transmission [55]. In contrast, high prevalence rates of *P. falciparum* infections (ranging from 37.6 to 39.7%) in Bloléquin and Odienné may imply that these districts are areas of high malaria transmission. This variation of *Plasmodium* transmission from one health district to another may be related to climatic conditions. Several previous studies have shown that *Plasmodium* infections are influenced by environmental factors such as temperature, rainfall, humidity and altitude [56, 57]. These factors directly or indirectly influence the development and appearance of *Anopheles* mosquitoes and, thus, the geographical distribution of the malaria infection and disease. The heterogeneity of malaria transmission observed in the present study is consistent with previous studies [27, 39]. This may also be the effect of differences in intervention strategies coordinated by the National Malaria Control Programme (NMCP), especially mass distribution of LLINs to vulnerable population and the use of ACT for the early treatment of malaria cases.

The country experienced already four campaigns of LLINs distribution and malaria transmission remains high and heterogeneous across cross-border health districts of Côte d’Ivoire with the presence of several vector species: *An. funestus s.l., An. nili* and *An. gambiae s.l.* The latter is the most species encountered. The study showed a high diversity and abundance of *Anopheles* mosquitoes, which could contribute to malaria transmission persistence over time. Moreover, high sporozoite infection and parity rates were recorded in all four heath districts and highlighted the high transmission of malaria within local populations. Currently, the vector control strategy of NMCP of Côte d'Ivoire is based on LLIN distribution. Recently in August 2020, the President Malaria Initiative (PMI) through the MoH implemented IRS in pilot site (Sakassou), in order to strengthen LLINs effects that have already proven effective in individual and community protection against malaria. [58, 59]. However, in the recent past, pyrethroids were the single insecticide class used for impregnation of LLINs, owing to their rapid action, excito-repellent effects, effectiveness at low doses and low toxicity to humans [60]. Unfortunately, pyrethroid resistance in malaria vectors has emerged and spread rapidly in Côte d’Ivoire [61] and several parts of Africa [62–64]. Therefore, efforts should be made to evaluate the effectiveness of insecticide-treated LLINs with different modes of action to which there is no cross-resistance and to evaluate promising tools to be used in combination with LLINs in highly endemic areas. Since mosquito capture has been done indoors, it is important to rapidly roll out the implementation of IRS nationally in highly endemic districts using next generation formulations, which has already proven its effectiveness in combination with LLIN [65].

## Conclusion

The specific diversity and abundance of anopheline vectors were high in the cross-border health districts of Côte d'Ivoire. *Plasmodium* sporozoite infection rates were also high, leading to continued and high transmission of malaria in local populations. *Anopheles gambiae s.l.* was the main malaria vector, with *An. funestus* playing a secondary vector role in the health districts of Bloléquin and Odienné. Malaria transmission remained high in the health districts of Odienné and Bloléquin where two additional vectors were involved, compared to the districts of Aboisso and Ouangolodougou. *Anopheles coluzzii* was most common in forest areas while *An. gambiae s.s.* predominated in the savannah areas of Côte d'Ivoire. Therefore, it is necessary to regularly monitor the bionomics of local *Anopheles* vector species and follow up possible change in malaria transmission dynamics to strengthen national vector control strategies taking into account the cross-border regions of Côte d'Ivoire. These observations may not only inform future research perspectives but should hopefully also guide future decision-making on malaria control strategies, especially in these cross-border areas of Côte d’Ivoire.

## Data Availability

All data generated or analysed during this study are included in this article and are available from the corresponding author.

## Abbreviations

ACT: Artemisinin-based combination therapy
COX-I: Cytochrome oxidase I
CSRS: Centre Suisse de Recherches Scientifiques
DNA: Deoxyribonucleic acid
EDTA: Ethylenediaminetetraacetic acid
IRS: Indoor residual spraying
KW: Kruskal–Wallis
LLINs: Long-lasting insecticidal nets
MoH: Ministry of Health
NMCP: National Malaria Control Programme
PCR: Polymerase chain reaction
PR: Parity rate
PSC: Pyrethrum spray collection
SINE-PCR: Short interspersed nuclear element-based polymerase chain reaction
TBE: Tris borate EDTA
UV: Ultraviolet
WET: Window exit traps
